# Chemical Modification of Glycoproteins’ Carbohydrate Moiety as a General Strategy for the Synthesis of Efficient Biocatalysts by Biomimetic Mineralization: The Case of Glucose Oxidase

**DOI:** 10.3390/polym13223875

**Published:** 2021-11-10

**Authors:** Marija D. Stanišić, Nikolina Popović Kokar, Predrag Ristić, Ana Marija Balaž, Milan Senćanski, Miloš Ognjanović, Veljko R. Đokić, Radivoje Prodanović, Tamara R. Todorović

**Affiliations:** 1 Faculty of Chemistry, University of Belgrade, Studentski trg 12-16, 11000 Belgrade, Serbia; mstanisic@chem.bg.ac.rs (M.D.S.); nikolina@chem.bg.ac.rs (N.P.K.); predrag@chem.bg.ac.rs (P.R.); rprodano@chem.bg.ac.rs (R.P.); 2 Institute of Chemistry, Technology and Metallurgy, National Institute of the Republic of Serbia, University of Belgrade, Njegoševa 6, 11000 Belgrade, Serbia; anamarija.balaz@ihtm.bg.ac.rs; 3 Institute of Nuclear Sciences “Vinča”, National Institute of the Republic of Serbia, University of Belgrade, Mike Petrovića Alasa 12-14, 11000 Vinča-Belgrade, Serbia; sencanski@vin.bg.ac.rs (M.S.); miloso@vin.bg.ac.rs (M.O.); 4 Innovation Center of the Faculty of Technology and Metallurgy, University of Belgrade, Karnegijeva 4, 11000 Belgrade, Serbia; vdjokic@tmf.bg.ac.rs

**Keywords:** metal–organic frameworks, ZIF-8, biomimetic mineralization, biocomposites, biocatalysts

## Abstract

Zeolitic imidazolate framework-8 (ZIF-8) is widely used as a protective coating to encapsulate proteins via biomimetic mineralization. The formation of nucleation centers and further biocomposite crystal growth is entirely governed by the pure electrostatic interactions between the protein’s surface and the positively charged Zn(II) metal ions. It was previously shown that enhancing these electrostatic interactions by a chemical modification of surface amino acid residues can lead to a rapid biocomposite crystal formation. However, a chemical modification of carbohydrate components by periodate oxidation for glycoproteins can serve as an alternative strategy. In the present study, an industrially important enzyme glucose oxidase (GOx) was selected as a model system. Periodate oxidation of GOx by 2.5 mM sodium periodate increased negative charge on the enzyme molecule, from −10.2 to −36.9 mV, as shown by zeta potential measurements and native PAGE electrophoresis. Biomineralization experiments with oxidized GOx resulted in higher specific activity, effectiveness factor, and higher thermostability of the ZIF-8 biocomposites. Periodate oxidation of carbohydrate components for glycoproteins can serve as a facile and general method for facilitating the biomimetic mineralization of other industrially relevant glycoproteins.

## 1. Introduction

According to MarketsandMarkets™ Company Research, the global enzyme market is projected to grow from USD 5.9 billion in 2020 to USD 8.7 billion in the next six years, owing to a global need for sustainability, higher process efficiency, and green, environmentally friendly technology and industry [1]. Significant progress in protein engineering has led to enzymes with enhanced catalytic performance and stability. Yet, the implementation of either native or mutant enzymes for various industrial applications is still limited due to the lack of protein activity and stability in harsh conditions. The immobilization of enzymes on solid supports can be an essential tool for the further improvement of their practical performances. Metal–organic frameworks (MOFs), porous coordination polymers consisting of metal containing nodes and organic ligands linked through coordination bonds, are superior to other porous materials commonly used to immobilize enzymes (zeolites, mesoporous silica, macroporous polymers, etc.). MOFs have ultrahigh porosity and one of the best surface area properties amongst all the support materials because they are finely tunable and crystalline, thus exhibiting uniformity and long-range ordering [2,3]. MOFs are perfect hosts that can protect guest enzymes from inhospitable external environments, such as elevated temperature, organic solvents, or proteolytic enzymes.

There are three general approaches for the preparation of MOF-based enzyme composites: surface attachment, post-synthetic diffusion, and encapsulation [3,4,5,6,7]. Either a surface attachment can be made by physical adsorption, or an enzyme can be grafted onto the MOF’s surface (covalent or supramolecular attachment). In such MOF-based enzyme composites (termed enzyme-on-MOF), enzymes are attached to the MOF surface, thus remaining primarily unprotected. The most recent advances in the field of development of MOF-based enzyme composites by the post-synthetic immobilization technique (termed enzyme@MOF) emphasizes the importance of the hierarchical pore structure of presynthesized MOFs for successful enzyme immobilization. The mesopores and micropores of the hierarchical porous MOFs can accommodate enzymes and facilitate substrate and product diffusion, respectively [5,8,9]. The common shortcomings of this strategy include the high price of ligands’ components and the low yield of the obtained enzyme@MOF composites. Encapsulation is another useful immobilization technique where a biocomposite (termed enzyme@MOF) is formed by the direct mixing of the MOF components (i.e., metal ions and ligands) in the presence of the enzyme. In comparison to post-synthetic diffusion, encapsulation gives a nonuniform distribution of enzyme molecules throughout the composite (poly)crystals and can only be employed with MOFs that have mild synthetic conditions, such as a special subset of MOFs known as zeolitic imidazolate frameworks (ZIFs). However, the advantage of encapsulation over the other two techniques is the low price of ZIFs’ components, the simplicity of the synthetic procedure, and the fast reaction times.

The most studied MOF for the encapsulation of biological species has been zeolitic imidazolate framework-8 (ZIF-8), which consists of Zn(II) ions in a tetrahedral geometry, bridged by 2-methylimidazole (HmIM) ligands [10,11]. Several enzymes were successfully encapsulated in ZIF-8 via the one-pot encapsulation method (i.e., by direct mixing of soluble enzyme, zinc(II) salt, and HmIM). In this process, various additives can be employed to promote biocomposite formation [4]. However, if the reaction is conducted in water without additives, the method is known as biomimetic mineralization [4]. This method is named after a sophisticated phenomenon in nature called biomineralization. Namely, many living organisms are capable of producing biominerals, which are composites of inorganic and organic materials with a precisely controlled structure and morphology [12]. The major mineralized tissues (such as bone, teeth, shells, etc.) are composed of calcium phosphate and carbonate minerals with a complex macromolecular matrix of proteins, polysaccharides, and lipids [12]. The rigid molecular architecture of the ZIF-8 was found to form a protective coating around the biomacromolecules, offering improved stability to external environments [11]. In this way, the ZIF-8 shell mimics the protective function of a biomineralized exoskeleton.

Several parameters influence the formation, structure, and activity of the enzyme@MOF composites, especially those obtained from ZIF-8 building units by the biomimetic mineralization process. Namely, three polymorphs comprising Zn(II) and HmIM/mIM and having different network topologies have been identified: ZIF-8 with a sodalite (**sod**) topology, **dia**-Zn(mIM)_2_ with a diamond topology, and **kat**-Zn(mIM)_2_ with a katsenite topology [13,14,15]. In addition, the amorphous phase amorph-Zn(mIM)_2_ and polymorphs U12 and U14, with an unknown topology, are also identified [16]. Among the mentioned materials, only ZIF-8 with a **sod** topology has a less densely packed structure with relatively large pore volumes and is able to facilitate selective molecular transport from the external environment to the encapsulated biomacromolecule [16,17]. It was shown that the introduction of biomacromolecule enhances the ZIF formation kinetics without influencing the final topology [16]. However, high HmIM/Zn(II) ratios and high precursor concentrations favor the formation of the kinetic **sod** polymorph [4,16]. Enzyme surface chemistry is also an important parameter. The formation of nucleation centers and further biocomposite crystal growth is fully governed by the pure electrostatic interactions between the enzyme’s surface and the positively charged metal ions [17]. A recent study, where a chemical modification of surface amino acid residues of various proteins was done, points out that the enhancement of these electrostatic interactions by the chemical functionalization of a protein surface, thus making it more negative, leads to a rapid biocomposite crystal formation. This method is proposed as a general strategy for facilitating biomimetic mineralization [18]. Taking into account the above-mentioned factors, and the fact that currently most of the industrially important enzymes are produced in yeasts and fungi, where the surface glycosylation serves as a mean of protection of extracellular proteins produced by these organisms, a chemical modification of carbohydrate components can serve as an alternative strategy. In the present study, we selected as a model system an industrially important enzyme glucose oxidase (GOx), a glycoprotein with a carbohydrate content of more than 20%, which catalyzes the conversion of β-D-glucose and molecular oxygen to D-glucono-1,5-lactone (which hydrolyzes spontaneously to gluconic acid) and H_2_O_2_ [19]. We chemically modified the surface carbohydrate components of GOx by a periodate oxidation using 2.5 mM sodium periodate. Under the applied oxidation conditions, the negative charge on the enzyme molecule was increased, which further led to a rapid biocomposite formation. Furthermore, we showed that obtained biocomposites display a higher thermal stability and activity when compared with the native counterparts. We have shown that simple periodate oxidation of the surface carbohydrate parts can be a facile and general method for controlling the electrostatic potential of a glycoprotein, thus facilitating the biomimetic mineralization under standard conditions. This study may provide a blueprint for how to tailor the surface chemistry of glycoproteins and anticipates that such a facile one-pot enzyme immobilization strategy will open new avenues in developing industrially important biocomposite catalysts with superior catalytic properties.

## 2. Materials and Methods

### 2.1. Materials

GOx from *Aspergillus niger* was purchased from Sigma-Aldrich (St. Louis, MO, USA). Peroxidase from horseradish (HRP) and 2,2′-azino-bis(3-ethylbenzothiazoline-6-sulfonic acid) (ABTS) were purchased from AppliChem (Darmstadt, Germany). Sodium metaperiodate (NaIO_4_) was purchased from VWR Chemicals (Leuven, Belgium). Zinc acetate dihydrate (Zn(O_2_CCH_3_)_2_·2H_2_O) and D-glucose monohydrate were purchased from Lachner (Neratovice, Czech Republic), while HmIM was purchased from Sigma-Aldrich (St. Louis, MO, USA). Sodium dodecyl sulfate (SDS) was purchased from Serva (Heilderberg, Germany). All reagents were of analytical grade and were used as purchased. All solutions were prepared with distilled water.

### 2.2. Periodate Oxidation

GOx (in the final concentration of 1.5 mg/cm^3^) was oxidized with 2.5, 5, and 50 mM NaIO_4_ in 50 mM sodium acetate buffer (pH = 5.0). The mixture was incubated at 4 °C in the dark for 6 h. The excess of NaIO_4_ was removed by addition of glycerin (0.1 M) for 30 min. Oxidized glucose oxidase (oxGOx) was then dialyzed against distilled water for 24 h at 4 °C. SDS and native PAGE electrophoresis were performed according to the Laemmli protocol [20]. Acrylamide concentrations were 10% for the resolving gel and 4% for the stacking gel. Coomassie Brilliant Blue staining solution was used to visualize the protein bands.

### 2.3. Biomimetic Mineralization

The stock solutions of native GOx (1.5 mg/cm^3^) or periodate oxidized enzymes (2.5—oxGOx, 5—oxGOx, and 50—oxGOx) were mixed with HmIM (1.25 M) and Zn(O_2_CCH_3_)_2_·2H_2_O (0.25 M) in two different ways. Regardless of the mixing procedure, the final concentrations of the biocomposite precursors were 0.35 mg/cm^3^ for proteins (as determined by UV absorbance, vide infra), 1.00 M for HmIM, and 0.02 M for Zn(O_2_CCH_3_)_2_·2H_2_O. In the first way of mixing (MX1), the stock solution of proteins was mixed with the HmIM solution and 1 min afterwards the Zn(O_2_CCH_3_)_2_·2H_2_O solution was added. In the second way (MX2), the stock solution of proteins was mixed with the Zn(O_2_CCH_3_)_2_·2H_2_O solution and 1 min afterwards the HmIM solution was added. The final concentration of the reactants in all reaction mixtures were 0.35 mg/cm^3^ for proteins, 1.00 M for HmIM, and 0.02 M for Zn(O_2_CCH_3_)_2_. The reaction mixtures were homogenized on a magnetic stirrer for 30 min and then left still at room temperature for 12 h. The obtained precipitates were centrifuged at 6000 rpm for 10 min. The supernatants were collected, while precipitates were washed with distilled water three times. Biocomposites obtained by the MX1 procedure are labeled as GOx-MX1 and oxGOx-MX1, while biocomposites obtained by the MX2 procedure are labeled as GOx-MX2 and oxGOX-MX2. Yields: 41.9 mg (GOx-MX1), 76.9 mg (oxGOx-MX1), 45.3 mg (GOx-MX2), and 77.8 mg (oxGOx-MX2).

All precipitates were incubated in 10% *w*/*v* SDS solution for 30 min to remove proteins adsorbed on a surface. After incubation, precipitates were washed two times with 0.1 M sodium acetate buffer (pH = 5.5), two times with 96% *w/w* ethanol, and distilled water to remove surfactant.

### 2.4. Determination of Protein Concentration

Protein concentrations were measured by UV absorbance at 280 nm on UV-1800 Shimadzu spectrophotometer (Thermo Fischer Scientific, Waltham, MA USA) using a quartz cell with 1.0 cm path length. The extinction coefficients for GOx and oxGOx at 280 nm is 2.67 × 10^5^ M^−1^ cm^−1^ [21]. It was confirmed that 2-HmIM and SDS did not affect protein concentration measurements.

### 2.5. Activity Measurements

The activity was measured using a coupled ABTS enzymatic assay. Enzymes/biocomposites were dissolved/resuspended in the reaction mixture containing 2 mM ABTS, 2 IU/cm^3^ HRP, and 0.5 M D-glucose in 0.1 M sodium acetate buffer (pH = 5.5). The activity was determined by monitoring a change in absorbance at 405 nm using the extinction coefficient of 36.8 mM^−1^ cm^−1^ for oxidized ABTS. The control reactions did not show an increase in absorbance at 405 nm associated with an increase of HRP concentration or ZIF-8. The specific activity, referring either to the enzymes or biocomposites, is obtained as U mg_enzyme_^−1^, where 1 U of the activity is defined as an amount of enzyme that converts 1 µmol of D-glucose per 1 min at 25 °C.

Specific activity of 2.5 mM periodate oxidized GOx (115.7 U/mg) did not change significantly compared to the commercial enzyme (116.8 U/mg) that showed no changes in diffusion limitations.

### 2.6. Thermal Stability Measurements

Thermostability of soluble proteins, biocomposites washed with water, and biocomposites washed with 10% *w*/*v* SDS was determined by incubation in water at 65 °C for 1 h and specific activity measurement afterwards, as described above. The residual activity of the samples was determined as the ratio of specific activities after and before incubation at 65 °C.

### 2.7. Powder X-ray Diffraction (PXRD)

PXRD experiments were conducted on a Rigaku SmartLab X-ray diffractometer (Rigaku, Neu-Isenburg, Germany) in *θ*-*θ* geometry (i.e., the samples in a horizontal position) in parafocusing Bragg–Brentano geometry using D/teX Ultra 250 strip detectors in 1D standard mode with CuKα_1,2_ radiation source (*U* = 40 kV and *I* = 30 mA). The PXRD patterns were collected in 5–65° 2θ range, with a step of 0.01° min^−1^.

### 2.8. Scanning Electron Microscopy (SEM)

The morphology of all-solid-state samples was characterized using a Tescan MIRA3 XMU Field Emission Scanning Electron Microscope (FESEM) (Brno, Czech Republic) operated at an acceleration voltage of 20 kV. Before the analysis, the samples were coated with a thin layer of Au using a standard sputtering technique.

### 2.9. Zeta Potential Measurements

The zeta potentials of the samples were measured at 25 °C in disposable zeta cells (DTS 1070) on a NanoZS90 (Malvern, UK) device. Measurements were performed after one minute of equilibrium time at native pH.

## 3. Results and Discussion

### 3.1. Periodate Oxidation

To increase the binding affinity of proteins for an immobilization support by controlling the electrostatic interactions, amino acid modifications are commonly applied [18,22]. It was shown that the reaction of surface lysine residues of various proteins with succinic (or acetic) anhydride facilitates biomimetic mineralization by increasing the surface negative charge [18]. Hence, the surface electrostatic potential of a protein can be used as a good indicator of its ability to induce biomimetic mineralization [18]. Many of the industrially important enzymes belong to the group of glycoproteins, thus a chemical modification of carbohydrate components can serve as an alternative strategy. To test our hypothesis, we performed periodate oxidation of a model enzyme GOx and tested the induction of ZIF-8 encapsulation under mild biomimetic mineralization conditions.

**Figure 1:**
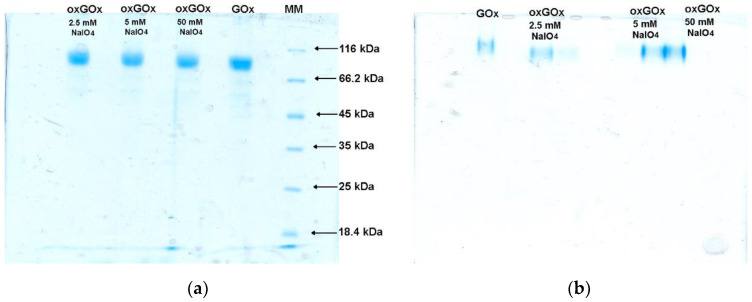
(**a**) SDS and (**b**) native PAGE electrophoresis of commercial GOx, periodate oxidized protein (oxGOx) using 2.5, 5, and 50 mM NaIO_4_. MM—molecular weight markers.

Chemical modification by periodate oxidation of GOx’s carbohydrate components was chosen because it was previously shown that the periodate oxidation did not alter the catalytic parameters, the gross structure, or the secondary and quaternary structures of the protein moiety [23,24]. We have performed periodate oxidation of GOx using NaIO_4_ in three different concentrations (2.5, 5, and 50 mM). The effect of the periodate oxidation was monitored by SDS and native PAGE electrophoresis (Figure 1). From the SDS electrophoresis (Figure 1a), it can be seen that periodate oxidation did not change the molecular weight of proteins. Thus, it can be assumed that periodate oxidation did not cause the deglycosylation of the protein. From the native PAGE electrophoresis (Figure 1b), an increase of the negative charge of all the oxidized samples can be observed.

Since the surface electrostatic potential of proteins is related to the zeta potential [18,25], we measured the zeta potential of commercial GOx and periodate oxidized samples (Table 1). These results are in line with the native PAGE electrophoresis, confirming an increase in the surface negative charge of periodate oxidized proteins. For further biomineralization studies, protein oxidized with 2.5 mM NaIO_4_ was chosen since the highest change in the zeta potential was noticed in this case (from −10.2 to −36.9 mV).

### 3.2. Biomimetic Mineralization Experiments

Next, we performed two sets of biomimetic mineralization experiments, considering previous findings that high HmIM/Zn ratios and high precursor concentrations favor the crystallization of the kinetic **sod** polymorph [16]. In all biomineralization experiments, the freshly prepared periodate oxidized GOx was used in order to avoid changes that can happen during the storage. In both sets of experiments, the following conditions were used: 0.35 mg/cm^3^ of a dissolved enzyme and a molar ratio of Zn^2+^:HmIM = 1:50. In the first set of biomimetic mineralization experiments (MX1), an aqueous solution of HmIM was mixed with an aqueous solution of enzymes (or water for control experiments), followed by the addition of an aqueous solution of zinc acetate. This sequence of addition of reactants was reversed in the second set of experiments (MX2), where an aqueous solution of zinc acetate was mixed with an aqueous solution of enzymes, followed by the addition of an aqueous solution of HmIM. The sequence of the addition of the reactants can be of importance, since in the first case the protein dissolution can be favored by the alkaline HmIM aqueous solution, and the formation of precipitates by Zn(II) ions is prevented, as discussed previously [16]. However, previous findings hypothesized that biomacromolecules concentrate positively charged zinc ions at their surface [17,18,26], thus promoting a more rapid biocomposite formation. Since oxGOx has the highest negative value of the zeta potential (Table 1), we wanted to check if the reversed addition of the reactants in the second set of biomineralization experiments can still have a high rapid biocomposite formation while avoiding the exposure of enzymes to extreme pH values of high HmIM concentration (pH = 11).

**Figure 2:**
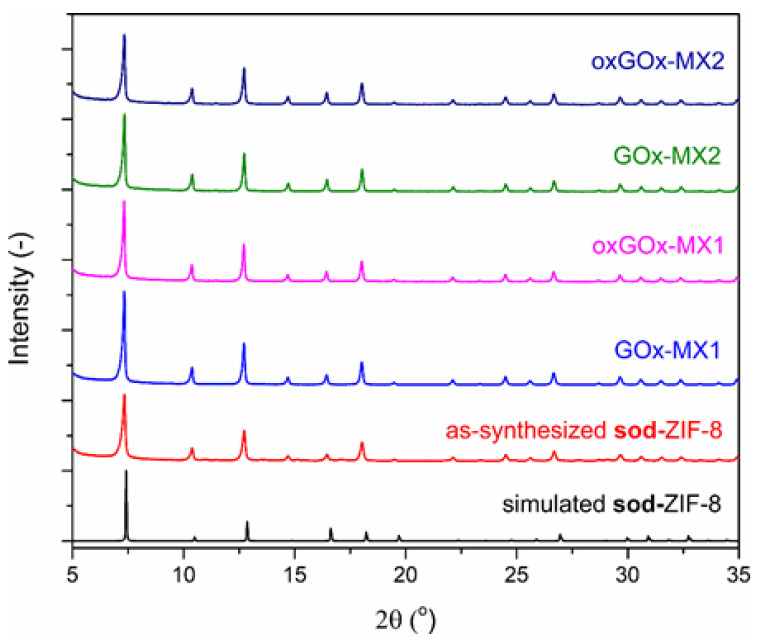
Simulated PXRD pattern of **sod**-ZIF-8 (black) and experimental PXRD patterns of as-synthesized **sod**-ZIF-8 (red), GOx-MX1 (blue), oxGOx-MX1 (magenta), GOx-MX2 (green), and oxGOx-MX2 (dark blue).

In the presence of enzymes, in both sets of biomineralization experiments, the PXRD results show that only **sod** polymorph was present in the obtained biocomposites (Figure 2).

**Figure 3:**
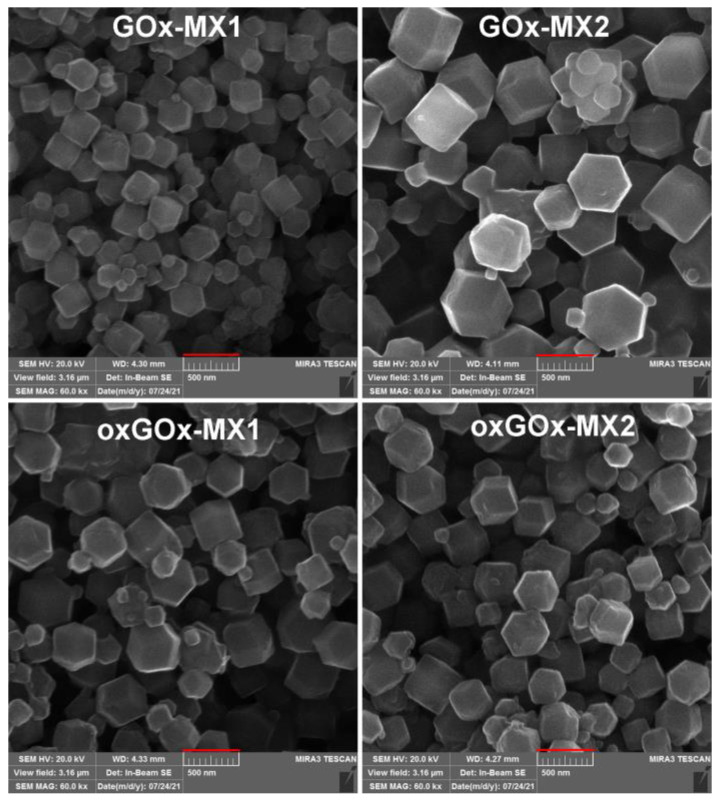
SEM images of biocomposites after washing with water (scale bar 500 nm).

The SEM images confirm the rhombic dodecahedral morphology of all the obtained biocomposites (Figure 3). This confirms that the applied synthetic conditions in all the cases led to the biocomposites of predetermined **sod** topology (i.e., high HmIM/Zn ratio and high concentration of reactants favor the crystallization of the kinetic **sod** polymorph) [16]. Interestingly, the four obtained biocomposites have different particle sizes (Figure 3). Classical and nonclassical crystallization theories have been extensively applied to understand the mechanism of the crystallization of biomimetic mineralization systems [27]. Previous findings suggest that controlled crystal formation and the size of crystalline particles are dependent on the formation of prenucleation clusters of ZIF-8 around the biomacromolecules [4]. However, further experiments are needed to explain the difference in particle size in our case.

As expected from the zeta potential measurements (Table 1), the yield of the oxGOx biocomposites was around two times higher than for the GOx composites (see the experimental part). The sequence of the addition of the reactants had a little influence on the yields, where the slightly higher yields were obtained when HmIM was added into the solution containing protein and Zn(II) salt.

### 3.3. Kinetic Immobilization Performance Parameters

MOF-based enzyme immobilization is an emerging multidisciplinary area that combines the principles of material science and protein biochemistry, and enzymology. Thus, for the assessment of these biocomposite materials, key parameters of each field should be carefully evaluated. This is important for the mechanistic understanding and the comparison of results among the various published studies. However, these comparisons are challenging due to the complex relationship between the enzyme, the MOF precursors, and the final structural properties of the MOF material. Moreover, the literature lacks a systematic evaluation of the “biochemical” properties of this class of biocomposites [4]. In the present study, we adopted important performance parameters pertaining to enzyme immobilization which were previously applied for other immobilization systems [4,28]. Two parameters describe the immobilization yield (*Y*) for activity (*A*) and protein (*P*) distributed between the liquid and the solid phase. Activity balance (*Y_A_*) is defined as the ratio of total immobilized enzyme activity (surface attached and encapsulated enzyme) and the activity of the enzyme used for the biomineralization experiment. Protein balance (*Y_P_*) is the ratio of the total immobilized amount of protein and the amount of protein used for the biomineralization experiment. Protein loading (*P*_loading_) represents mg of immobilized protein per one g of the carrier. The activity of the immobilized enzyme per unit mass of solid biocomposite is the specific activity of the biocomposite, while the specific activity of the bound enzyme is the activity of the immobilized enzyme per one mg of immobilized protein. The ratio of the specific activity of the bound enzyme and specific activity of the free soluble enzyme is the effectiveness factor (*η*).

Table 2 summarizes the key immobilization performance parameters for four types of biocomposites, washed only with water, and containing two types of enzymes (GOx or oxGOx) obtained in two sets of biomimetic mineralization experiments, while Table 3 contains data for the same samples washed additionally with 10% (*w*/*v*) SDS solution (vide supra). Control experiments with pure ZIF-8 showed that there was no interference with activity and protein concentration measurements.

From the obtained results, it can be seen that the key kinetic parameters were similar between all four samples after first washing with water (Table 2). However, after washing the samples with SDS in order to remove the surface adsorbed proteins, a drastic difference in kinetic performance was observed between the samples containing GOx and the samples containing oxGOx (Table 3). All the key parameters, like *Y_P_*, *P*_loading_, specific activities, and *η*, were significantly reduced in the case of the GOx samples. For example, *Y_P_* was not changed significantly after SDS washing in the case of the oxGOx samples (oxGOx-MX1-SDS: 0.50; oxGOx-MX2-SDS: 0.55; Table 3), while a significant drop of *Y_P_* was observed with the GOx samples (GOx-MX1-SDS: 0.28; GOx-MX2-SDS: 0.26; Table 3). These results are in alignment with *P*_loading_ data (Table 3). Moreover, the specific activity of the biocomposites, as one of the most important parameters, for the oxGOx-SDS samples are one order of magnitude higher than for the GOx-SDS samples (Table 3). Interestingly, the specific activities of the bound enzymes, and especially *η*, were also much higher in the case of the oxGOx-SDS samples when compared to the GOx-SDS samples (Table 3).

Regardless of the mixing procedure, our data (*Y_P_* and *P*_loading_, Table 2 and Table 3) show that almost half of the bound protein in the case of GOx is attached to the surface of the biocomposite, contrary to oxGOx, where only around 15% of the protein is surface attached. These results can be of importance since surface adsorbed proteins usually lead to the lower stability of biocatalysts.

Unfortunately, although there are few publications describing GOx@ZIF-8 biocomposites synthesis [29,30,31], due to different immobilization conditions (pH, temperature, concentrations, additives, etc.), and, more importantly, only partial information on key kinetic parameters, it is not possible to perform a meaningful comparison of the results. For example, Wu et al. showed that GOx can be encapsulated into ZIF-8 by one-pot immobilization reaction under conditions similar to ours in 30 min using zinc nitrate [30]. Obtained biocomposite polycrystals were additionally cross-linked with dopamine. The effectiveness factor of GOx@ZIF-8 was 1.5%, while for the cross-linked GOx@ZIF-8, the effectiveness factor was 0.7%. Protein loading was comparable to ours, but biocomposite was washed only with water. There are no data on specific activities.

### 3.4. Thermal Stability Study

**Figure 4:**
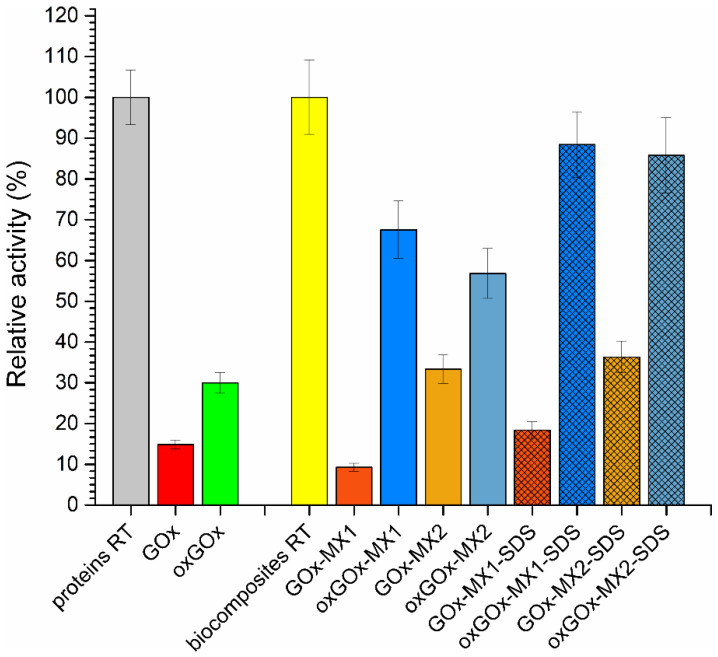
Thermal stability of free enzymes, biocomposites washed with water, and biocomposites washed with SDS. Relative activity of the samples after incubation in water at 65 °C for 1 h.

The thermostability of soluble enzymes and biocomposites was measured by incubation at 65 °C for 1 h. Results of the thermostability are depicted in Figure 4. The relative activities of the samples were expressed as a percentage of the original activity before the thermal treatment.

Native GOx and oxGOx retained no more than 30% of their original activity. In all four biocomposite samples before washing with SDS, the oxGOx biocomposites show higher stability compared to the GOx biocomposites. Additional washing of biocomposites with SDS, which removed surface adsorbed enzyme molecules, increased the thermostability of the enzyme biocomposites compared to the same samples before washing. This was the result of the higher stability of the enzyme molecules encapsulated within the biocomposite compared to the surface adsorbed ones that were removed by SDS. In all cases, the oxGOx biocomposites show two to four times higher thermostability compared to the GOx biocomposites. For example, the oxGOx-MX1-SDS retained 90% of activity after 1 h incubation at 65 °C, while the GOx-MX1-SDS retained only 20% of the activity after incubation at 65 °C. The obtained results of the thermal stability study are better than previously reported for GOx@ZIF-8 biocomposite [32].

**Figure 5:**
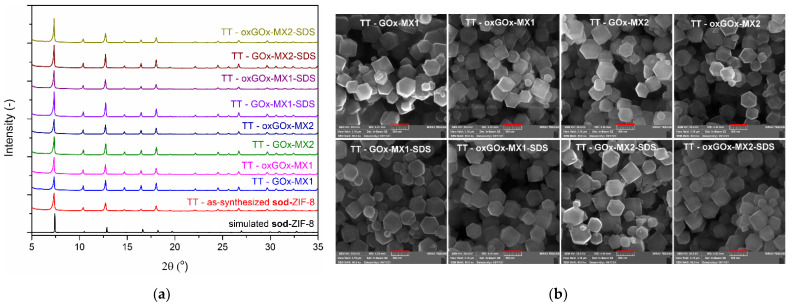
(**a**) PXRD diffractograms of the biocomposites washed with SDS after thermal treatment; (**b**) SEM images of the biocomposites washed with SDS after thermal treatment (scale bar 500 nm).

Thermal treatment did not influence the topology and morphology of the biocomposites, as shown by PXRD experiments and SEM, respectively (Figure 5). These results clearly show that biomineralization with periodate oxidized GOx resulted in biocomposites with increased specific activities and significantly increased thermostability of the encapsulated enzyme.

## 4. Conclusions

In this work, we have proved that the chemical modification of the carbohydrate moiety of the protein molecules by periodate oxidation can significantly influence the yield, the key kinetic parameters, and the thermostability of the ZIF-8 biocomposites. The oxidation of GOx increased negative charge on the enzyme molecule from −10.2 mV for commercial GOx to −36.9 mV for 2.5 mM periodate oxidized GOx, as shown by the zeta potential measurements and native PAGE electrophoresis. This increase was probably due to the formed carboxylate groups within the carbohydrate moiety of the glycoprotein. Consequently, biomineralization experiments with oxGOx resulted in a higher specific activity, effectiveness factor, and the higher thermostability of the ZIF-8 biocomposites. Further, the oxGOx biocomposites washed with SDS retained a high specific activity and an increased thermostability when compared to SDS-treated GOx biocomposites, most likely due to the better encapsulation efficiency (~0.5% for oxGOx samples vs. ~0.25% for GOx samples). These results clearly show that the carbohydrate part of protein molecules have a significant influence on the properties and the formation of enzyme@MOF biocomposites by biomimetic mineralization. This may be of high importance for other industrially relevant glycoproteins like HRP, CALB, LiP, etc., that are produced extracellularly.

## Figures and Tables

**Table 1:** Experimental zeta potential of the tested protein solutions ^1^.

Sample	Zeta Potential (mV)
GOx	−10.2 ± 4.6
2.5-oxGOx	−36.9 ± 5.6
5-oxGOx	−32.7 ± 4.4
50-oxGOx	−29.5 ± 5.2
GOx in Zn(II) solution	−1.95 ± 3.8
GOx in HmIM solution	−13.1 ± 3.6
2.5-oxGOx in Zn(II) solution	−1.93 ± 4.2
2.5-oxGOx in HmIM solution	−23.6 ± 4.9

^1^ 0.35 mg/cm^3^ of enzymes in distilled water, 0.02 M Zn(II) or 1.00 M for HmIM solutions.

**Table 2:** Key immobilization performance parameters for four types of biocomposites, washed only with water.

Parameter	GOx-MX1	oxGOx-MX1	GOx-MX2	oxGOx-MX2
*Y*_A_	0.82	0.99	0.95	0.99
*Y*_P_	0.52	0.56	0.40	0.63
*P*_loading_ (mg/g_carrier_)	41.25	24.14	37.47	36.56
Specific activity (U/g_biocomposite_)	1591.25	855.84	815.02	873.51
Specific activity (U/mg_enzyme bound_)	39.18	25.61	33.51	28.83
*η* × 100 (%)	22	22	19	24

**Table 3:** Key immobilization performance parameters for four types of biocomposites, washed with water and additionally with 10% (*w*/*v*) SDS solution.

Parameter	GOx-MX1-SDS	oxGOx-MX1-SDS	GOx-MX2-SDS	oxGOx-MX2-SDS
*Y*_A_	0.28	0.50	0.26	0.55
*Y*_P_	24.40	28.90	22.72	32.72
*P*_loading_ (mg/g_carrier_)	52.55	534.55	15.88	338.90
Specific activity (U/g_biocomposite_)	2.32	20.46	2.45	22.59
Specific activity (U/mg_enzyme bound_)	1.3	17	3	19
*η* × 100 (%)	0.28	0.50	0.26	0.55

## Data Availability

The data presented in this study are available upon request to the corresponding author.
